# Hypermutation and microsatellite instability in gastrointestinal cancers

**DOI:** 10.18632/oncotarget.22783

**Published:** 2017-12-01

**Authors:** Kizuki Yuza, Masayuki Nagahashi, Satoshi Watanabe, Kazuaki Takabe, Toshifumi Wakai

**Affiliations:** ^1^ Division of Digestive and General Surgery, Niigata University Graduate School of Medical and Dental Sciences, Chuo-ku, Niigata City, Niigata 951-8510, Japan; ^2^ Department of Respiratory Medicine and Infectious Diseases, Niigata University Graduate School of Medical and Dental Sciences, Chuo-ku, Niigata City, Niigata 951-8510, Japan; ^3^ Breast Surgery, Department of Surgical Oncology, Roswell Park Cancer Institute, Buffalo, NY 14263, USA; ^4^ Department of Surgery, University at Buffalo Jacobs School of Medicine and Biomedical Sciences, The State University of New York, Buffalo, NY 14203, USA

**Keywords:** hypermutation, microsatellite instability, immune checkpoint inhibitor, gastrointestinal cancer, precision medicine

## Abstract

Recent progress in cancer genome analysis using next-generation sequencing has revealed a high mutation burden in some tumors. The particularly high rate of somatic mutation in these tumors correlates with the generation of neo-antigens capable of eliciting an immune response. Identification of hypermutated tumors is therefore clinically valuable for selecting patients suitable for immunotherapy treatment. There are several known causes of hypermutation in tumors, such as ultraviolet light in melanoma, tobacco smoke in lung cancer, and excessive APOBEC (apolipoprotein B mRNA editing enzyme, catalytic polypeptide-like) activity in breast and gastric cancer. In gastrointestinal cancers, one of the leading causes of hypermutation is a defect in DNA mismatch repair, which results in microsatellite instability (MSI). This review will focus on the frequency, characteristics and genomic signature of hypermutated gastrointestinal cancers with MSI. Detection of tumor hypermutation in cancer is expected to not only predict the clinical benefit of immune checkpoint inhibitor treatment, but also to provide better surgical strategies for the patients with hypermutated tumors. Thus, in an era of precision medicine, identification of hypermutation and MSI will play an important role directing surgical and chemotherapeutic treatment.

## INTRODUCTION

Surgery is the most effective treatment for localized gastrointestinal cancer, and is often curative. Advanced stage cancers, however, are difficult to control with surgery alone. In these cases, surgery with the addition of multidisciplinary treatment strategies, such as combined chemoradiotherapy and molecular targeted therapy, can be beneficial. For example, combination chemotherapy with cetuximab for unresectable colorectal cancer (CRC) liver metastases refractory to conventional chemotherapy, increased resection rates and improved patient outcomes [1]. More recently, the emergence of immune checkpoint inhibitors has brought about a paradigm shift in cancer treatment. These have had dramatic effects in several advanced solid cancers [2–4], and accumulating evidence suggests promising outcomes in advanced gastrointestinal cancers [5]. Importantly, in some patients, immune checkpoint inhibitors can provide a cure for metastatic cancer, which is beyond the ability of conventional surgical treatment.

**Figure 1:**
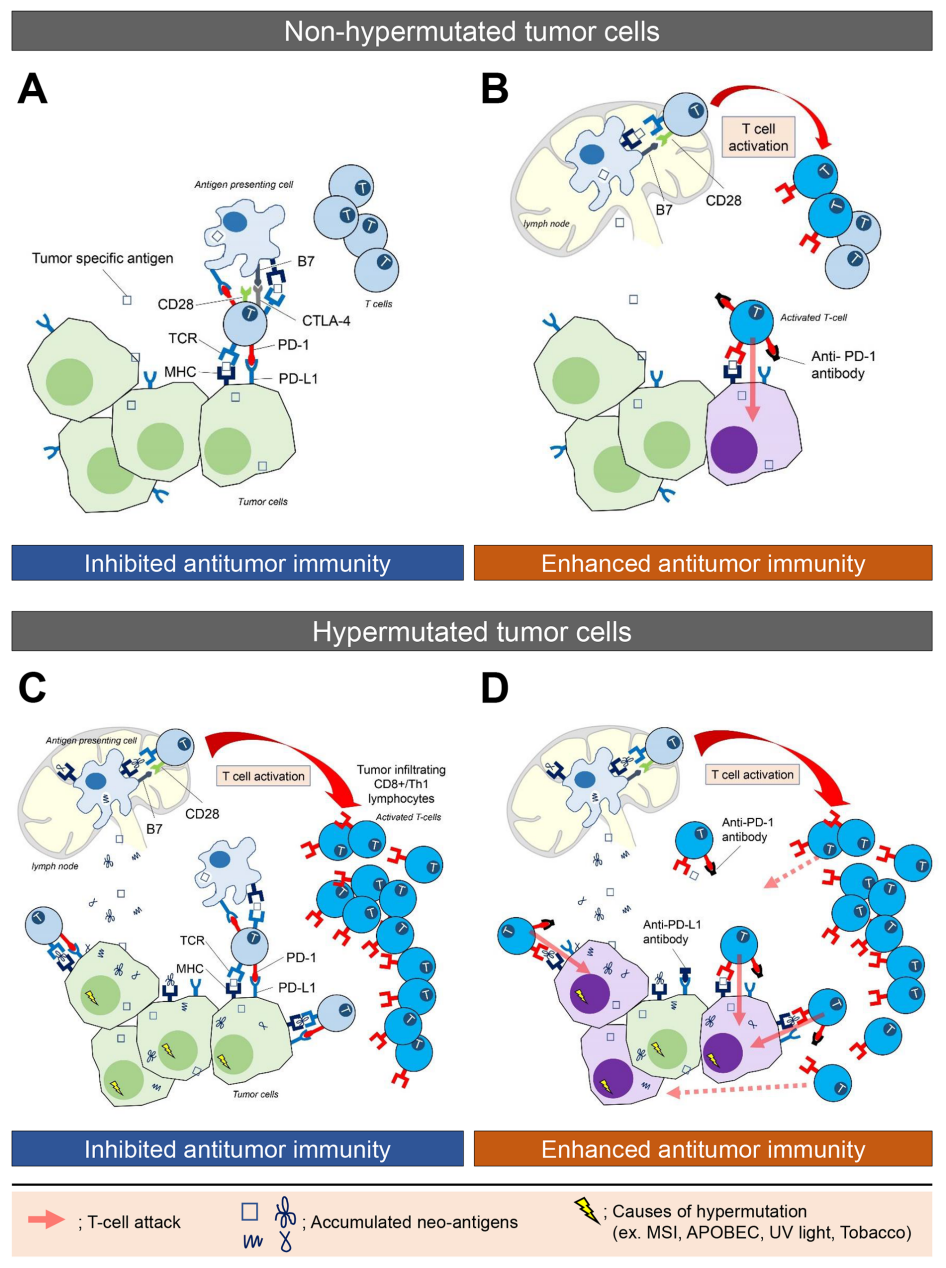
The immune microenvironment in non-hypermutated and hypermutated tumors, and enhanced immune activity following blockade of the PD-L1/PD-1 interaction **(A)** Tumor-specific antigens are processed and presented by cancer cells and antigen presenting cells (APCs). Upregulated expression of checkpoint molecules, including PD-1 and CTLA-4 on T cells and PD-L1 on tumor cells and APCs, delivers inhibitory signals that suppress T cell activation, and produce an immunosuppressive microenvironment. **(B)** Blockade of the PD-L1/PD-1 interaction by an anti-PD-1 antibody enhances immune activity, which leads to T cells attacking and killing tumor cells. The attacked tumor cells are shown as purple cells. **(C)** Hypermutated cancer cells, derived by various mutagenic processes, generate numerous neo-antigens (mutated proteins) that are processed and presented by cancer cells and APCs. This stimulates T cell activation, leading to an infiltration of cytotoxic (CD8+) T-lymphocytes. Checkpoint molecules inhibit antitumor activity. **(D)** Blockade of the PD-L1/PD-1 interaction by an anti-PD-1 antibody enhances immune activation, whereby the infiltrating cytotoxic (CD8+) T-lymphocytes and activated Th1 cells attack tumor cells presenting the tumor specific antigen. This is thought to explain why hypermutated tumors demonstrate a significant durable efficacy to immune checkpoint therapy.

Immunotherapy, however, is only effective in a small proportion of patients, and current methods cannot identify which tumor is likely to respond. Predictive biomarkers are therefore needed to assist oncologists identify candidates for whom this therapeutic approach is most likely to succeed. Recent progress in genomic analysis using next-generation sequencing (NGS) technology has enabled comprehensive detection of mutations and mutation burden in cancer tissues. A hypermutated tumor is defined as a tumor with an increased mutation burden (a high rate of somatic mutation). The threshold above which tumors are considered hypermutated, however, depends on the sequencing methodology and type of cancer (Table 1). Importantly, the clinical significance of identifying hypermutated tumors has recently been demonstrated by several studies showing tumor mutation burden correlates with the generation of neo-antigens (mutated proteins) and a clinical response to immunotherapy [6, 7] (Figure 1). The causes of hypermutation vary between cancer types (Table 2). Ultraviolet (UV) light is the cause of many mutations in melanoma [8], while tobacco smoke causes the mutations that accumulate in non-small cell lung cancer [6]. A leading cause of the mutations found in several gastrointestinal cancers, such as colorectal, gastric, and hepato-pancreato-biliary cancer, is dysfunction in the mismatch repair (MMR) system. Indeed, CRC patients with MMR deficiency, who would be expected to develop a hypermutated phenotype, exhibited excellent outcomes after anti-PD-1 therapy [5]. This highlights the clinical significance of identifying hypermutated tumors for immunotherapy treatment.

**Table 1:** Definitions of hypermutated tumors reported in the literature

Cancer	Sequence method	Mutation rate	Reference
Colorectal	WES	>12 per Mb	[16]
Stomach	WES	>11.4 per MB	[50]
Stomach	WES and WGS	20.5 per Mb	[113]
Biliary tract	WES and transcriptome sequencing	>11.13 per Mb	[87]
Endometrium	WES	>18 per Mb	[114]
Melanoma	WES	>100 per exome	[10]
Lung	WES	≧178 nonsynonymous mutation per tumor	[6]
Glioblastoma	WES	>100 per tumor exome	[115]
Glioma	Targeted NGS	>20 per 1.4 Mb	[116]

WES, whole-exome sequencing; WGS, whole-genome sequencing; NGS, next-generation sequencing.

**Table 2:** The various causes of hypermutation in different cancers

Cause of hypermutation	Organ	Reference
UV light	Skin cancer	[8]
Tobacco smoke	Lung cancer	[6]
MSI	Gastrointestinal cancer	[16]
APOBEC	Breast cancer	[15]
POLE, POLD1	Colorectal cancer	[21, 40, 41]

UV, ultraviolet; MSI, microsatellite instability; APOBEC, apolipoprotein B mRNA editing enzyme, catalytic polypeptide-like; POLE, DNA polymerase E; POLD1, DNA polymerase D1.

In this article, we review the current understanding of hypermutation and MMR deficiency in gastrointestinal cancer from the perspective of surgical oncology. We discuss how new knowledge of the cancer genome can be best used to improve the treatments available for patients with gastrointestinal cancer.

## HYPERMUTATION IN CANCER AND ITS CAUSES

Mutations in oncogenes and tumor suppressor genes are the main mechanisms for cancer development. Increased spontaneous or environmentally enhanced mutagenesis has been correlated with increased mutation load and cancer risk [9]. Importantly, hypermutated cancer cells are believed to create numerous neo-antigens, which promote infiltration of cytotoxic (CD8+) T-lymphocytes and activated Th1 cells to the tumor microenvironment (Figure 1). Furthermore, studies have indicated an association between elevated mutation burden and response to checkpoint blockade immunotherapy in certain solid cancers [5, 6, 10, 11].

Hypermutation in cancer can be caused by a variety of mechanisms, including exogenous mutagens and endogenous processes. Exposure to exogenous mutagens, such as UV light in melanoma or tobacco smoke in lung cancer [12], can cause an accumulation of mutations in affected cells. Endogenous mutagenic processes can also affect the number of mutations [13]. They include activation-induced deaminase in chronic lymphocytic leukemia and B-cell lymphomas [14], and excessive APOBEC (apolipoprotein B mRNA editing enzyme, catalytic polypeptide-like) activity in breast and gastric cancers [15]. One of the leading causes of hypermutation in gastrointestinal cancer is defective DNA MMR systems, which results in microsatellite instability (MSI) [16].

## DETERMINING MSI STATUS AND MMR DEFICIENCY

MSI is a well-established tumorigenesis pathway that refers to the hypermutable state of cells. It is caused by a dysfunction of the MMR system, which results in a reduction in the length of highly repeated DNA sequences termed microsatellites. The MMR system corrects nucleotide mismatches that occur during replication. Microsatellites are simple repeat sequences of one to six base pairs (also known as short tandem DNA repeats) that are prone to DNA replication errors, resulting in MSI [17]. In sporadic CRCs, MMR deficiency most commonly occurs through epigenetic inactivation from hypermethylation of the *MLH1* gene [16]. A germline mutation inactivating one of the MMR genes (*MLH1*, *MSH2*, *MSH6*, and *PMS2*) may lead to a hereditary form, termed hereditary non-polyposis colon cancer (HNPCC) [18].

MSI status is commonly determined by polymerase chain reaction amplification. DNA is isolated from microdissected tumor and normal tissues to compare the length of microsatellite alleles. The National Cancer Institute (NCI) recommends a microsatellite panel (NCI panel) consisting of two mononucleotide repeats (*BAT25* and *BAT26*) and three dinucleotide repeats (*D5S346*, *D2S123*, and *D17S250*) [17]. Using the NCI panel, microsatellite instability-high (MSI-H) tumors are defined as having instability in two or more markers, and tumors with low or stable MSI have instability in one or no markers. Tumor MMR status is determined by immunohistochemical analysis of the proteins encoded by genes involved in DNA MMR. MSI-H tumors are found not only in colorectal cancer patients, but also in other gastrointestinal cancer patients, with the frequency of their occurrence differing between each cancer type (Table 3).

**Table 3:** The frequency of microsatellite instability (MSI-H) in each gastrointestinal cancer

Cancer	Frequency of MSI-H	Reference
Colorectal cancer	12-17 %	[17, 23–25]
Gastric cancer	8-37 %	[43, 46–49]
Hepatocellular carcinoma	0-18 %	[68–72]
Pancreatic cancer	0-13 %	[76–81]
Intrahepatic cholangiocarcinoma	10 %	[90]
Gallbladder cancer	0-42 %	[90, 95–99]
Ampullary carcinoma	0-22 %	[90, 96, 101–107]

Recently, several groups have described NGS as a suitable testing platform for MSI [19–21]. The advantage of NGS technology is that it allows massively parallel sequencing capable of producing millions of sequences at once [22]. This usually translates to more efficient genetic sequencing compared to traditional genetic testing platforms, which is beneficial when testing large batches of tumor samples [20].

## MSI IN PATIENTS WITH CRC

MSI is often reported in patients with CRC, and is detected in about 15% of all CRCs [17, 23–25]. Of these, 3% are associated with Lynch syndrome, also known as HNPCC, and the remaining 12% are sporadic, caused by acquired hypermethylation of the *MLH1* promoter [25]. Hypermutated CRC is highly correlated with MSI. The Cancer Genome Atlas (TCGA) Network has reported that 16% of CRCs are hypermutated (defined as cancers with mutation rates of >12 per 10^6^ bases), with three-quarters of these having high MSI [16]. One of the most frequent genetic alterations in CRCs with MSI is the oncogenic *BRAF* V600E mutation. The TCGA study has also revealed that hypermutated CRCs had fewer *APC*, *KRAS*, and *TP53* mutations compared with the non-hypermutated CRCs. In contrast, mutations in transforming growth factor (TGF)-β signaling genes and *BRAF* were dramatically elevated in the hypermutated tumors.

Regardless of the origin (hereditary or sporadic) or type of mutation, MSI-H CRCs share some distinct histopathological cancer features. For example, these tumors tend to arise in the proximal or right side, and are more common in females [26]. This is particularly true in sporadic MSI-H cancers where over 90% are located in the proximal colon [27, 28]. It has been reported that CRCs with MSI-H, especially tumors with *BRAF* mutations, show increased proliferative activities [29]. Histologically, MSI-H CRCs tend to be poorly differentiated, are often mucinous, and sometimes contain signet ring cells and undifferentiated medullary carcinoma. Pathological characteristics are associated with the presence of lymphocytic infiltration [30, 31], and a Crohn’s-like lymphocytic reaction. Indeed, excessive lymphocyte infiltration was classically used as a pathological screening criterion for MSI by hematoxylin and eosin staining.

Clinically, MSI-H CRC develops a large size tumor with high levels of cell growth but less metastasis [30, 32]. MSI-H CRC patients are reported to have a good prognosis [33] and it has been suggested they respond differently to chemotherapy than microsatellite stable (MSS) tumor patients. For example, MSI-H patients have been reported to be less likely to respond to fluoropyrimidine and 5-fluorouracil (5-FU) [33, 34]. This finding remains controversial, however, as several reports have indicated there is no significant clinical benefit in using MSI status to guide treatment decisions on the use of 5-FU for CRC [35]. Furthermore, 5-FU, alone or in combination with other drugs, has been the standard of care for first-line treatment in Stage III, Stage IV, and high-risk Stage II CRC since the late 1950s.

MSI tumors strongly express various immunological checkpoint proteins, such as PD-1, PD-L1, CTLA-4, LAG-3 and IDO [36–39], and, as such, are the focus of many current clinical trials. Their inhibitory signals prevent elimination of neoplastic cells by counteracting the active immune microenvironment of the MSI tumor [5, 36]. Recently, it has been reported that CRC patients with MSI showed significantly better progression-free and overall survival than patients without MSI. Therefore, the MSI status and hypermutated phenotype may be a predictive marker for immuno-modulating agents [25].

In addition to dysfunction in the MMR system, mutations in DNA polymerase D1 (*POLD1*) and DNA polymerase E (*POLE*) genes have been described as another cause of hypermutated CRC [40, 41]. In addition to somatic mutations, germline mutations in these genes have been identified in familial CRC. POLD1 synthesizes the lagging strand and POLE1 synthesizes the leading strand in a bidirectional replication fork [42]. CRC with mutations in the exonuclease domain of *POLE1* is associated with a high number of mutations, multiple tumor neo-epitopes, and extensive T lymphocyte infiltration. Taken together, a hypermutated phenotype, not only with MSI-H, but also with a *POLE* mutation, may be a useful predictive marker for CRC.

## MSI IN PATIENTS WITH GASTRIC CANCER

The MSI-H phenotype in gastric cancer is predominantly caused by epigenetic hypermethylation of *MLH1* rather than germline mutations in an MMR gene [39, 43–45]. The incidence of MSI-H in gastric cancers varies from 8-37% [43, 46–49]. TCGA has defined four molecular subgroups of gastric cancer by unsupervised clustering, and one of the groups, comprising 22% of all cases, was enriched for MSI and showed elevated mutation rates and hypermethylation [50]. Gastric cancer with MSI-H is reported to display distinct clinical and molecular features compared to MSS gastric cancer [46, 51–53]. They are usually associated with female sex, older age [54, 55], antral location, intestinal type [49], smaller risk in lymph node metastasis [49, 55], shallower tumor invasion [49], earlier stage, and a better prognosis [52, 53, 55], most of which are characteristics similar to CRC MSI-H patients, as described above. Association with tumor necrosis, expanding growth pattern, and tumor-infiltrating lymphocytes (TILs) are also reported [56]. Although reports are inconsistent [47, 56–58], MSI in gastric cancer may be considered a favorable prognostic indicator [52, 58] for both early [53, 59–61] and advanced [46, 49] stages.

Conflicting results have been reported in MSI-H gastric cancers regarding response to adjuvant 5-FU-based chemotherapy. No difference in overall survival between MSI-H and MSS was observed in a study of 240 patients [39]. However, a more recent study found disease-free survival was improved in the MSI-Low/MSS group treated with 5-FU-based chemotherapy [62]. The disparity between these studies might be due to the high morphological, phenotypic, and molecular heterogeneity of gastric cancer [56]. Recently, significant correlations have been found between defective MMR systems and immune system activity, suggesting that this group of patients might be optimal candidates for immunotherapies including anti-PD-1/PD-L1 antibodies [63–65].

Mutational analysis of MSI-H gastric cancers revealed 37 significantly mutated genes, including *TP53*, *KRAS*, *PIK3A*, and *AR1D1A* [50]. Genes in the TGF-β pathway were predicted to be key drivers in MSI. Indeed, *TGFBR2*, *ACVR2A*, *SMAD4*, and *ELF3* are frequently mutated, suggesting an important role in gastric cancer biology [66, 67].

## MSI IN PATIENTS WITH HEPATO CELLULAR CARCINOMA (HCC) AND PANCREATIC CANCER

MSI is seldom observed in HCC and pancreatic carcinoma. MSI-H occurrence in HCC ranges from 0-18% [68–72], and alterations in MMR genes are not implicated in its pathogenesis [73–75]. As such, the biological and clinicopathological significance of MSI in HCC remains to be determined [74]. In a small-sized study, the histology and the prognosis of patients with MSI-H HCC were worse than those with non-MSI-H HCC [71]. MSI-H tumors tended to exhibit a large, unique nodule without a capsule, corresponding to a more aggressive tumor [71]. Progression of the primary tumor leading to liver failure is the general cause of death in HCC patients, rather than tumor metastasis. Considering MSI-H HCC patients exhibit a more aggressive primary tumor than those with non-MSI-H, it is to be expected that MSI-H HCC patients have a worse prognosis. In contrast, in other cancer types such as CRC, gastric cancer and other gastrointestinal cancers, prognosis is usually determined by metastatic disease rather than primary tumor characteristics. As such, MSI-H patients with these cancer types often exhibit a better prognosis than non-MSI-H patients, likely due to the lower frequency of metastasis in MSI-H cancer.

MSI is rarely found in sporadic pancreatic ductal adenocarcinoma, occurring in less than 1% of cases based on molecular MSI testing [76]. Studies that used the NCI panel to define MSI-H found it occurred in 0-13% of pancreatic carcinoma [77–80]. A recent study of 385 pancreatic cancers subjected to whole genome or exome sequencing reported four (1%) cases with MSI [81]. MSI is found in both medullary [77, 82, 83] and non-medullary [79, 84, 85] subtypes of pancreatic carcinoma. Previous studies on MSI as a prognostic predictor of pancreatic cancer are limited. Some reports suggest that MSI-H pancreatic cancer might have a comparatively better prognosis than non-MSI-H cancer [77, 86], but larger studies are needed to confirm this finding.

## MSI IN PATIENTS WITH BILIARY TRACT CANCER (BTC)

Published literature on the mutational profile and MMR deficiency in BTCs is limited, but there are reports that some BTCs have a significantly high mutation burden [87, 88]. Most studies evaluating MSI in intrahepatic cholangiocarcinoma come from analyses of patients in Southeast Asia, particularly Thailand, where liver cholangiocarcinoma represents one of the most common cancers and is believed to be associated with liver fluke (*Opisthorchis viverrini*) infection [89, 90]. In these studies, the frequency of MSI-H in intrahepatic cholangiocarcinoma was 10% [90]. Liver-fluke-associated intrahepatic cholangiocarcinoma shows a higher somatic mutation burden compared with non-parasite-associated BTC [91].

Recent studies from Japan have investigated mutational signatures of intrahepatic cholangiocarcinoma believed to be caused from exposure to organic solvents, mainly haloalkanes such as 1,2-dichloropropane and/or dichloromethane [92–94]. The number of single-nucleotide variants in cholangiocarcinoma resected from printing workers exposed to organic solvents was significantly higher than in control common cholangiocarcinoma tissues, with somatic mutations at an average of 44.8/Mb [92]. This suggests 1,2-dichloropropane is an exogenous mutagen that results in hypermutated cholangiocarcinoma.

Previous reports have found a correlation between MSI and gallbladder carcinoma. The prevalence of MSI varied from 0% to 42%, and averaged 5% overall [90, 95–99]. There was no significant difference in tumor stage or overall survival between patients with and without MSI [100], and no association between MSI status and tumor grade, or the presence of extracellular mucin or TILs [100]. Expression of long interspersed nuclear element-1 (LINE-1), a surrogate marker of global methylation status, was lacking in MSI gallbladder carcinomas, suggesting the loss of MMR proteins was due to changes in methylation [100]. MSI was found, not only in cancer regions, but also in severe chronic cholecystitis [97], and areas of both intestinal metaplasia and dysplasia [98]. This suggests that MMR deficiency in tumor development may be associated with prolonged inflammation and may occur very early in gallbladder cancer development [90, 97, 98].

Several studies have correlated ampullary carcinoma with MMR deficiency [90, 101–106], while others have reported that MSI could not be identified in ampullary carcinoma [96, 107]. The reported frequency of MSI-H in ampullary carcinomas is 0-22%. Ampullary and colorectal carcinomas share a significant overlap in phenotypic and molecular characteristics [106]. Ampullary carcinoma with MSI-H often demonstrates better prognosis [101, 105] with increased TILs [104, 105], poor differentiation with “medullary”-like histology, and intestinal type morphology [104, 105, 108].

Most recently, a relatively large-scale study by Nakamura et al. [87] has molecularly characterized 260 BTCs and uncovered a spectrum of genomic alterations. Fourteen cases were classified as hypermutated, with mutation rates of >11.13/Mb. Of these, five harbored inactivating (nonsense, frameshift or splice-site) mutations in mismatch-repair complex components. Transcriptome sequencing and hierarchical clustering of gene expression levels classified BTC into four molecular subgroups that had prognostic implications. The hypermutated cases were significantly enriched in the worst prognosis group, with increased expression of immune checkpoint molecules and enrichment for the genes involved in cytokine activity and anti-apoptosis. In total, 45% of cases showed increased expression of immune checkpoint molecules, which suggests that this subgroup may be a good target population for immunotherapy [87, 88, 109].

## FUTURE PERSPECTIVE AND CONCLUSION

While immunotherapy and targeted therapies continue to advance the treatment of cancer, surgery still plays a vital role in the treatment of gastrointestinal malignancies, especially in the management of early-stage, localized disease. However, despite surgery with curative intent in patients with advanced disease, many experience tumor recurrence that leads to poor outcome [110]. The genomic profile of the primary and metastatic tumor provides critical information for guiding decisions about treatment. This review has outlined how hypermutation may play a pivotal role as a cancer biomarker able to identify cancer subtypes most likely to respond to treatment, and therefore predict the clinical benefit of immunotherapy, especially in advanced cancers where surgery is usually not indicated.

BTCs are heterogeneous cancers with an increasing incidence worldwide. They are often refractory to standard chemotherapy regimens and exhibit a poor prognosis. Recent studies have revealed that BTCs are rich in actionable genetic aberrations [111]. As such, it is now possible to identify unique molecular subsets of BTC, such as hypermutated phenotypes, that can be effectively treated with a personalized medicine approach, which will hopefully lead to an improved prognosis [111].

Detection of hypermutation in cancers may not only predict the clinical benefit of immune checkpoint inhibitors, but has the potential to also provide better surgical strategies for the patients with hypermutated tumors. Of particular benefit may be the treatment of locally advanced hypermutated tumors. Hypermutated tumors tend to show expansive growth in a localized region and have less metastasis. As such, surgical resection, rather than the currently indicated neoadjuvant chemotherapy, may prove more beneficial. Furthermore, MSI-H tumors tend to show resistance to 5-FU-based chemotherapy compared to non-MSI-H tumors. Therefore, a combination of surgery and immune checkpoint inhibitors would be an attractive approach for cancer treatment. This approach, however, needs further investigation since studies of combined immunotherapy and surgery are lacking. Immune checkpoint inhibitors are now being incorporated in various clinical trials in the neoadjuvant setting (NCT02957968, NCT02735239, NCT03003637, NCT02918162) [112], the adjuvant setting (NCT02775812, NCT02641093), or both (NCT02296684). These studies will provide valuable evidence of any clinical benefit from immune checkpoint inhibitor use combined with surgery.

## Abbreviations

5-FU: 5-fluorouracil
APOBEC: apolipoprotein B mRNA editing enzyme, catalytic polypeptide-like
BTC: biliary tract cancer
CD: cluster of differentiation
CRC: colorectal cancer
CTLA-4: cytotoxic T-lymphocyte-associated antigen 4
HCC: hepatocellular carcinoma
HNPCC: hereditary non-polyposis colon cancer
IDO: indoleamine 2,3-dioxygenase
LAG-3: lymphocyte activation gene-3
LINE-1: long interspersed nuclear element-1
MMR: mismatch repair
MSI: microsatellite instability
MSI-H: microsatellite instability-high
MSS: microsatellite stable
NCI: The National Cancer Institute
NGS: next-generation sequencing
PD-1: programmed death-1
PD-L1: programmed death ligand 1
POLD1: DNA polymerase D1
POLE: DNA polymerase E
TCGA: The Cancer Genome Atlas
TGF: transforming growth factor
Th1: T helper cell type 1
TILs: tumor-infiltrating lymphocytes
UV: ultraviolet
